# A Phosphorylation-Related Variant *ADD1*-rs4963 Modifies the Risk of Colorectal Cancer

**DOI:** 10.1371/journal.pone.0121485

**Published:** 2015-03-27

**Authors:** Na Shen, Cheng Liu, Jiaoyuan Li, Xueqin Chen, Yang Yang, Ying Zhu, Yajie Gong, Jing Gong, Rong Zhong, Liming Cheng, Xiaoping Miao

**Affiliations:** 1 Department of Epidemiology and Biostatistics, and the Ministry of Education Key Lab of Environment and Health, School of Public Health, Tongji Medical College, Huazhong University of Science and Technology, Wuhan, Hubei, China; 2 Department of Laboratory Medicine, Tongji Hospital, Tongji Medical College, Huazhong University of Science and Technology, Wuhan, China; Medical College of Soochow University, CHINA

## Abstract

It is well-established that abnormal protein phosphorylation could play an essential role in tumorgenesis by disrupting a variety of physiological processes such as cell growth, signal transduction and cell motility. Moreover, increasing numbers of phosphorylation-related variants have been identified in association with cancers. ADD1 (α-adducin), a versatile protein expressed ubiquitously in eukaryotes, exerts an important influence on membrane cytoskeleton, cell proliferation and cell-cell communication. Recently, a missense variant at the codon of ADD1’s phosphorylation site, rs4963 (Ser586Cys), was reported to modify the risk of non-cardia gastric cancer. To explore the role of *ADD1*-rs4963 in colorectal cancer (CRC), we conducted a case-control study with a total of 1054 CRC cases and 1128 matched controls in a Chinese population. After adjustment for variables including age, gender, smoking and drinking, it was demonstrated that this variant significantly conferred susceptibility to CRC (G versus C: OR = 1.16, 95% CI = 1.03–1.31, P = 0.016; CG versus CC: OR = 1.25, 95% CI = 1.02–1.55, P = 0.036; GG versus CC: OR = 1.35, 95% CI = 1.06–1.72, P = 0.015). We further investigated the interaction of *ADD1*-rs4963 with smoking or drinking exposure, but found no significant result. This study is the first report of an association between *ADD1* and CRC risk, promoting our knowledge of the genetics of CRC.

## Introduction

Colorectal cancer (CRC), representing approximately 10% of the global incidence of cancer, ranks fourth among human cancers worldwide [1,2]. In China, it is estimated that almost 253,000 new cases occur per year, representing an upward trend these years [1,3]. Several environmental risk factors have been reported to be associated with CRC [4–7], such as smoking, drinking and obesity. However, only a few exposed individuals ultimately develop CRC, and familial aggregation exists [8], implying that genetic components also have an important role in CRC risk.

Recent explosions of candidate gene researches and genome-wide association studies (GWAS) have greatly promoted our understanding of genetic components of CRC and identified several loci across the whole genome, but these findings can explain only a portion of the susceptibility. Accordingly, there is a need to identify more potential variants to fill the blanks of CRC genetic architecture. Protein phosphorylation is one of the most important post-translational modifications existing ubiquitously in eukaryotes; it regulates almost 30% of all cellular proteins and plays an essential role in many physiological processes such as signal transduction, energy metabolism and cellular plasticity [9–11]. Previous studies have shown that abnormal phosphorylation is associated with complex diseases including colorectal cancer [12–14]. Moreover, increasing observations of functional experiments have suggested that non-synonymous single nuclear polymorphisms (nsSNPs) could play a role in tumorgenesis by directly or indirectly influencing protein phosphorylation [15–19]. For example, Luna, et al. found that the *hOGG1* Ser326Cys polymorphism (rs1052133) could disturb the nucleolar localization of this protein by changing its phosphorylation status in the cell cycle; this functional variant has been reported to modify the risk of a variety of cancers [15]. More recently, Chellappa, et al. investigated the relationship between Src kinase and HNF4α in human colon cancer and also found that three functional variants harbored in HNF4α (rs6093980, rs6031602 and rs1063239) could increase phosphorylation by Src kinase and decrease the stability of HNF4α protein [19]. All this accumulated evidence strongly suggests that phosphorylation-related variants could affect pathogenesis and might be a good cut point to explore the potential causal variants of CRC risk.

Adducin, consisting of α- with β- or γ-subunits, is an actin-binding protein that plays a crucial role in the assembly of the membrane cytoskeleton and cell-cell adhesion. The activity of this protein is regulated by phosphorylation via several classic kinases such as protein kinase A (PKA), protein kinase C (PKC) and Src kinase [20,21]. The essential component, α- adducin, is encoded by the *ADD1* gene and is expressed ubiquitously. In 2003, Bowen, et al. reported that the expression of *ADD1* was up-regulated in tumors and accompanied by increased expression of Ki67, linking this protein to cell proliferation [22]. Subsequently, Chen, et al. found that the depletion of α- adducin could affect cell-cell junctions and cell proliferation [23]. A recent study further found that phosphorylated ADD1 also exerted an important function during mitosis [24].

Interestingly, Wang, et al. have recently combined data from the PhosphoSitePlus and dbSNP databases to screen phosphorylation-related variants across the *ADD1* gene region, and they have identified only one missense variant with minor allele frequency (MAF) greater than 0.05 in the Han Chinese population, namely, rs4963 (Ser586Cys) [25]. This C/G variant produces the amino acid substitution of cysteine for serine at codon 586, and the phosphoserine has been detected by previous studies [26,27]. Wang, et al. performed a case-control study with 1998 cases and 2008 controls and found that *ADD1*-rs4963 (MAF = 0.496) might be associated with susceptibility to non-cardia gastric cancer (CG versus CC: OR = 1.24, 95% CI = 1.06–1.46, *P* = 0.008; GG versus CC: OR = 1.49, 95% CI = 1.25–1.78, *P* < 0.001) [25]. Informatively, we hypothesized that *ADD1*-rs4963 might also contribute to CRC risk by affecting the phosphorylation of ADD1. Hence, a case-control study including 2182 subjects was conducted in a Chinese population to examine this hypothesis.

## Materials and Methods

### Study subjects

This case-control study recruited 1054 primary CRC patients and 1128 cancer-free controls. All these subjects were unrelated Han Chinese. Cases were pathologically confirmed and consecutively recruited between June 2009 and February 2013 from Tongji Hospital in Wuhan, central China. Patients who had a history of any other cancer or tumor were excluded. Controls were randomly selected from people who participated in health examinations in the Wuhan area during the same period during which cases were recruited. A portion of these controls have been described in our previous studies [28–32]. The inclusive criteria for controls were that the subjects should be cancer-free and frequency matched to cases by age (±5 years) and gender. During recruitment, blood samples and demographic information were obtained from each participant after he/she signed an informed consent voluntarily. Definitions of smoking and drinking exposures were detailed previously [28]. Our study was performed with the approval of the Tongji Medical College’s institutional review boards from Huazhong University of Science and Technology.

### Genotyping assay

Genomic DNA was extracted from anticoagulant peripheral blood of each subject using RelaxGene Blood DNA System (TIANGEN, Beijing, China). *ADD1*-rs4963 was genotyped by the TaqMan SNP Genotyping Assay (Life technologies, Carlsbad, California) via 7900HT Fast Real-Time PCR System (Life technologies, Carlsbad, California).Negative controls and 5% duplicates were monitored for quality control. In this study, the concurrence rate of the duplicates was 100%. The genotyping call rate for *ADD1*-rs4963 was 98.4%.

### Statistical analysis

A goodness-of-fit χ^2^ test was used to examine the Hardy-Weinberg equilibrium (HWE) for genotype of *ADD1*-rs4963 in controls (*P* = 0.808). Differences between cases and controls were evaluated by the independent-samples t test or Pearson’s χ^2^ test in the distribution of demographic characteristics. The association of *ADD1*-rs4963 with CRC risk was estimated with the odds ratio (OR) and corresponding 95% confidence interval (95% CI), using unconditional logistic regression (LR) analysis without or with adjustment for gender, age, smoking and drinking. The potential gene-environment interaction between *ADD1*-rs4963 and smoking or drinking was assessed by the genotype-smoking or genotype-drinking combined effect and the multiplicative interaction term. A two-sided *P* < 0.05 was used as the criterion of statistical significance. All the analyses were performed with PASW Statistics 18.0 (IBM Corporation, New York). In addition, a study power of 0.94 was estimated via Power v3.0 [33,34] to detect an OR of 1.35 given our sample size.

## Results

### Characteristics of study subjects

A total of 1054 CRC cases and 1128 cancer-free controls were included in this case-control study, and their characteristics are demonstrated in Table 1. We did not observe any significant difference between cases and controls in age (*P* = 0.388), gender (*P* = 0.576) or smoking status (*P* = 0.201). However, significantly more drinkers were observed among the cases than among the controls (33.2% versus 28.3%, *P* = 0.013).

**Table 1:** Characteristics of participants in this case-control study.

	Case (n = 1054)	Control (n = 1128)	*P*
	N (%)	N (%)	
Age (mean ± SD)	59.43 ± 11.72	58.97 ± 12.78	0.388
Gender			0.576
Male	623 (59.1)	680 (60.3)	
Female	431 (40.9)	448 (39.7)	
Smoking status			0.201
Non-smokers	699 (66.3)	777 (68.9)	
Smokers	355 (33.7)	351 (31.1)	
Drinking status			0.013
Non-drinkers	704 (66.8)	809 (71.7)	
Drinkers	350 (33.2)	319 (28.3)	

Abbreviations: SD, standard deviation.

### Association between *ADD1*-rs4963 and CRC risk

The association analyses of *ADD1*-rs4963 with CRC risk are summarized in Table 2. The allele frequency differed significantly between cases and controls (*P* = 0.011). A similar result was also shown for the genotype distribution (*P* = 0.028). After adjustment for age, gender, smoking and drinking, an increased risk of CRC was statistically associated with CG (OR = 1.25, 95% CI = 1.02–1.55, *P* = 0.036) and GG genotype (OR = 1.35, 95% CI = 1.06–1.72, *P* = 0.015) compared with CC genotype. In the dominant model, CG and GG individuals showed a 1.28-fold higher risk than CC individuals based on multifactorial logistic analysis (OR = 1.28, 95% CI = 1.05–1.57, *P* = 0.014). Moreover, the G allele demonstrated more susceptibility to CRC, with an adjusted OR of 1.16 (95% CI = 1.03–1.31, *P* = 0.016) under the additive model.

**Table 2:** Association analyses between *ADD1*-rs4963 and CRC risk.

	Case	Control	Crude OR (95% CI)	*P*	Adjusted OR^a^ (95% CI)	*P* ^*a*^
	N (%)	N (%)				
Allele				0.011		0.016
C	992 (47.6)	1135 (51.5)	Reference		Reference	
G	1094 (52.4)	1071 (48.5)	1.17 (1.04–1.32)		1.16 (1.03–1.31)	
Genotype				0.028		0.038
CC	229 (22.0)	294 (26.7)	Reference		Reference	
CG	534 (51.2)	547 (49.6)	1.25 (1.02–1.55)	0.035	1.25 (1.02–1.55)	0.036
GG	280 (26.8)	262 (23.8)	1.37 (1.08–1.75)	0.010	1.35 (1.06–1.72)	0.015
Recessive				0.100		0.141
CC+CG	763 (73.2)	841 (76.2)	Reference		Reference	
GG	280 (26.8)	262 (23.8)	1.18 (0.97–1.43)		1.16 (0.95–1.41)	
Dominant				0.011		0.014
CC	229 (22.0)	294 (26.7)	Reference		Reference	
CG+GG	814 (78.0)	809 (73.3)	1.29 (1.06–1.58)		1.28 (1.05–1.57)	
Additive				0.011		0.016
CC	229 (22.0)	294 (26.7)	Reference		Reference	
CG	534 (51.2)	547 (49.6)	1.17 (1.04–1.32)		1.16 (1.03–1.31)	
GG	280 (26.8)	262 (23.8)				

^a^ Adjusted for variables including age, gender, smoking and drinking status.

In addition, we performed interaction analyses between *ADD1*-rs4963 and smoking or drinking exposure, but found no significant result (Table 3).

**Table 3:** Analyses of interaction between *ADD1*-rs4963 and environmental exposure.

	OR^a^ (95% CI)		*P* ^b^ for interaction	OR^c^ (95% CI)		*P* ^d^ for interaction
	non-smokers	smokers		non-drinkers	drinkers	
CC	Reference	1.07 (0.71–1.60)	0.952	Reference	1.26 (0.84–1.90)	0.563
CG	1.24 (0.96–1.60)	1.36 (0.98–1.89)		1.19 (0.93–1.53)	1.79 (1.28–2.48)	
GG	1.37 (1.02–1.83)	1.40 (0.94–2.07)		1.38 (1.03–1.84)	1.65 (1.13–2.42)	
CC	Reference	1.07 (0.71–1.60)	0.991	Reference	1.27 (0.84–1.91)	0.668
CG+GG	1.28 (1.01–1.63)	1.37 (1.01–1.86)		1.25 (0.99–1.58)	1.74 (1.29–2.36)	
CC+CG	Reference	1.09 (0.85–1.41)	0.744	Reference	1.43 (1.10–1.84)	0.414
GG	1.18 (0.94–1.50)	1.21 (0.85–1.72)		1.23 (0.97–1.56)	1.47 (1.04–2.06)	

^a^ Data were adjusted for age, gender and drinking status.

^b^ *P* values for *ADD1*-smoking interaction were calculated with the multiplicative interaction LR model.

^c^ Data were adjusted for age, gender and smoking status.

^d^ *P* values for *ADD1*-drinking interaction were calculated with the multiplicative interaction LR model.

## Discussion

Our research reported here is based on a case-control study performed with a total of 1054 CRC cases and 1128 matched controls to explore the association of *ADD1*-rs4963 with susceptibility to CRC in a Chinese population. Our results demonstrated that the G allele significantly increased the CRC risk compared with the C allele for this variant. Previous research has also consistently shown that the G allele is the risk allele associated with non-cardia gastric cancer [25]. To the best of our knowledge, the current study is the first report of a relationship between *ADD1* and CRC risk.

Protein phosphorylation, usually occurring on serine, threonine and tyrosine residues, turns the function or activity of many proteins on and off, enabling the effect control of cellular life processes including cell growth, differentiation and apoptosis. The marked increase of studies in the genomic era has resulted in the identification of many nsSNPs across the human genome. Although they represent a very small proportion of the known human variants (<1%), these nsSNPs could alter amino acids, affect post-translational modifications, and influence the stability and function of proteins, thus playing a role in the pathogenesis of complex diseases. In 2006, Erxleben, et al. first proposed “phosphorylopathy” to describe the human variants that contribute to aberrant protein phosphorylation [35]. Later, Yang, et al. reported 64 known disease-related phosphorylation sites by integrating information on nsSNPs from dbSNP with information on phosphorylation sites from experimental data [36]. Ryu, et al. performed a genome-wide prediction of genetic variants that affect phosphorylation sites or kinases [37]. Recently, Ren, et al. developed the PhosSNP database to help researchers screen important nsSNPs affecting protein phosphorylation [38]. In addition to findings in silico, experimental and epidemiological studies have also identified increasing numbers of phosphorylation-related variants involved in tumorgenesis, including CRC [15–19,25]. Variants involving protein phosphorylation have been attracting increasing attention.

*ADD1*, located at 4p16.3, encodes α- adducin, which is expressed ubiquitously in a variety of tissues and which is an important component of the membrane cytoskeleton, modifying cellular mitosis, adhesion, and communication [20–24]. It is well-known that phosphorylation plays a key role in regulating the function of this protein. For example, phosphorylation of Ser716 of ADD1 has been found to suppress importin α to bind to the nuclear localization signal (NLS) of α-adducin, hence physiologically preventing the nuclear translocation of this protein; however, the Ser716Ala mutant primarily accumulated in the nucleus and was rarely exported to the cytoplasm to participate in the formation of adhesions, thereby influencing cell-cell communication [23]. Previous studies have suggested that *ADD1* potentially plays a role in tumors [22–24]. Specially, Wang, et al. reported an association of *ADD1*-rs4963 (Ser586Cys) with the risk of non-cardia gastric cancer [25]. The *ADD1*-rs4963 C allele is at the codon of TCT encoding serine, whereas the G allele is at the codon of TGT encoding cysteine. Several studies have identified the phosphoserine at codon 586 of ADD1 [26,27]. When the C→G mutation occurs, the serine residue will change into a cysteine residue, and this site can not be phosphorylated due to a lack of hydroxyl groups. In addition, this variant belongs to the COOH-terminal tail of α- adducin (amino acid 430–737), which is a region often regulated by kinases [23,24]. Thus, we speculated that the G risk allele of *ADD1*-rs4963 might affect the phosphorylation of α- adducin and then disturb its activity, including cellular proliferation and communication, thus affecting the development of CRC.

Several limitations should be acknowledged here. First, only one variant in or near *ADD1* gene was genotyped in our study. That was because *ADD1*-rs4963, located at an experimentally verified phosphorylation site, was the only candidate variant identified by Wang, et al. by combining data from the PhosphoSitePlus and dbSNP databases [25]. We agreed that it was interesting to evaluate other variants in or near *ADD1* gene, which helped to determine whether this variant was “real” one in this region associated with CRC. We will try to investigate in our future studies. Second, this was a case-control study. The retrospective observational design inevitably causes two main types of bias, one is selection bias due to non-randomization, and another is information bias such as recall bias. Thus our participants could not be perfectly representative of the whole population. Third, data on certain other risk factors including family history, obesity and ulcerative colitis were not fully collected during the investigation, with the result that the ORs of CRC risk could not be adequately adjusted to estimate the risk effect of *ADD1*-rs4963. In addition, *ADD1*-rs4963 was not located at the susceptibility loci identified by CRC GWAS. A possible explanation is that GWAS data require more stringent significance level (*P* < 5×10^−8^) for analysis, thus usually leaving out many potential susceptibility loci. Unfortunately, we failed to get the original CRC GWAS data for further examination. We fully understand our limitations and more studies are wanted to verify our results.

In summary, the current study highlighted that the phosphorylation-related variant *ADD1*-rs4963 could confer CRC risk. This finding further advances our understanding of the genetics of this disease. Independent replication studies and experiments in functional biology are still needed to verify our result and further investigate the mechanism underlying the association.
